# A novel method of assessing quality of postgraduate psychiatry training: experiences from a large training programme

**DOI:** 10.1186/1472-6920-13-85

**Published:** 2013-06-14

**Authors:** Mukhtar Bizrah, Eduardo Iacoponi, Elizabeth Parker, Janice Rymer, Amy Iversen, Simon Wessely

**Affiliations:** 1 Guy’s & St Thomas’ Hospitals NHS Foundation Trust, King’s College London, London, UK; 2 Division of Medical Education, King’s College London, London, UK; 3 South London and Maudsley NHS Foundation Trust, London, UK; 4 Department of Psychological Medicine, Institute of Psychiatry, King’s College London, London, UK; 5 Department of Psychological Medicine, Weston Education Centre, 10 Cutcombe Road, Denmark Hill, London, Post code: SE5 9RJ, UK

**Keywords:** Postgraduate Training, Postgraduate Medical Education, Psychiatry Training, Non-anonymised interviews, Non-anonymised feedback, Training quality, Trainees Feedback, Trainer Feedback

## Abstract

**Background:**

Most assessments of the quality of postgraduate training are based on anonymised questionnaires of trainees. We report a comprehensive assessment of the quality of training at a large postgraduate psychiatry training institute using non-anonymised face-to-face interviews with trainees and their trainers.

**Methods:**

Two consultant psychiatrists interviewed 99 trainees and 109 trainers. Scoring of interview responses was determined by using a pre-defined criteria. Additional comments were recorded as free text. Interviews covered 13 domains, including: Clinical, teaching, research and management opportunities, clinical environment, clinical supervision, adequacy of job description, absence of bullying and job satisfaction. Multiple interview domain scores were combined, generating a ‘Combined’ score for each post.

**Results:**

The interview response rate was 97% for trainers 88% for trainees. There was a significant correlation between trainee and trainer scores for the same interview domains (Pearson’s r = 0.968, p< 0.001). Overall scores were significantly higher for specialist psychiatry posts as compared to general adult psychiatry posts (Two tailed *t*-test, p < 0.001, 95% CI: -0.398 to −0.132), and significantly higher for liaison psychiatry as compared to other specialist psychiatry posts (*t*-test: p = 0.038, 95% CI: -0.3901, -0.0118). Job satisfaction scores of year 1 to year 3 core trainees showed a significant increase with increasing seniority (Linear regression coefficient = 0.273, 95% CI: 0.033 to 0.513, ANOVA p= 0.026).

**Conclusions:**

This in-depth examination of the quality of training on a large psychiatry training programme successfully elicited strengths and weakness of our programme. Such an interview scheme could be easily implemented in smaller schemes and may well provide important information to allow for targeted improvement of training. Additionally, trends in quality of training and job satisfaction amongst various psychiatric specialities were identified; specifically speciality posts and liaison posts in psychiatry were revealed to be the most popular with trainees.

## Background

Recruitment and retention of psychiatry trainees is an established problem in many countries around the world, including the UK, USA and Australia [1-3]. Numerous studies in the literature have sought to assess quality of training in psychiatry, degree of job satisfaction, trainee perspectives on active problems in psychiatry training or potential reasons for leaving the psychiatry training pathway [4-7]. In the UK, for example, the National Trainee Survey [8] is completed by large numbers of clinical trainees, and asks about satisfaction and experiences of the training experience in a rotation (e.g. first core psychiatry training year), rather than specific feedback for an individual post (e.g. post X for which there is a named supervisor). As such, the existing surveys generate anonymous results on ‘blunt’ measures of trainee experience which can only be interpreted at the level of a scheme. This is useful but gives little information about what may be going on within a rotation, nor does it identify areas of weakness or conversely good practice [9]. Nevertheless, despite these weaknesses, studies such as the National Trainee Survey have assumed great importance in improving quality of training provision [10].

In this paper, we report an interview-based assessment method conducted to comprehensively assess the quality of psychiatry training in a large postgraduate psychiatry training scheme. We believe this to be the first such study that uses non-anonymised face-to-face interviews of pairs of trainees and their consultant trainers to collect individualised feedback on specific posts, as opposed to a broad ‘snapshot’ of an entire scheme. This enabled us to collect comprehensive feedback, as well as enabling a direct comparison of the perceptions of both parties at the level of each individual placement. The objective of the assessment described in this paper was to guide future improvements in overall training provision on this large rotation as well as to quality assure individual placements that trainees undertake. In this paper, we discuss only the results obtained from trainees and their trainers which are generalisable for other psychiatry training programmes.

## Methods

### Setting

The Maudsley Training Scheme is the largest Psychiatry Speciality training programme in the UK [11], and we suspect, the largest in the world. The South London and Maudsley NHS Foundation Trust (SLaM) forms the majority of the scheme [12]. It provides training to 125 out of 150 core psychiatry training posts and to 94 higher psychiatry trainees, although only the former are the subject of this paper. SLaM serves a population of approximately one million residents in the four London boroughs of Lambeth, Southwark, Croydon and Lewisham, each of which has an Emergency Department at which SLaM provides mental health services. Each post lasts six months and is supervised by a named trainer, who must be a consultant psychiatrist.

### Interviewees

**Figure 1:**
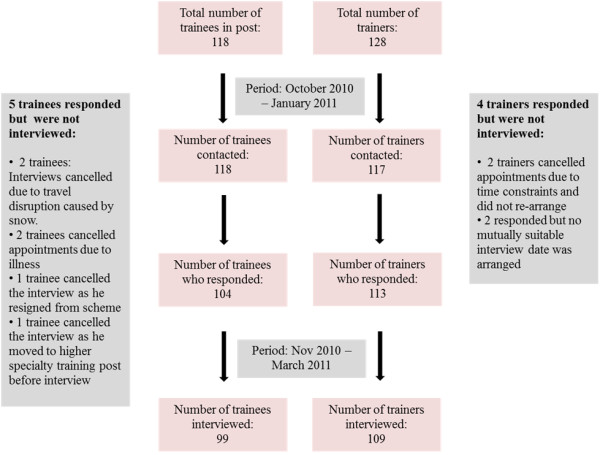
Total number of trainees and trainers who were contacted, responded and interviewed.

Interviews were conducted with core psychiatry trainees and their consultant trainers. Each trainee or trainer was interviewed separately. Figure 1 summarises the interview scheme timeline. The response rate was 88% and 97% for trainees and trainers respectively.

### Contacting trainers and trainees

The Medical Director of the Trust and the Vice Dean for Education and Training emailed all trainers about the aims of the current study and encouraged participation. The two consultant interviewers sent out a similar email to all trainees. All trainers and trainees were then sent an email offering 2–3 potential interview slots at their place of work. This email included the interview questions and the respective job descriptions. A reminder email was then sent out a month later with alternative appointment times.

### Areas covered by interview

The interview included:

1) Questions from the General Medical Council (GMC) 2010 National Training Survey [13].

2) Additional questions covered by previous local trainee surveys carried out at SLaM.

Table 1 shows a summary of the areas covered by questions to trainees and their trainers.

**Table 1:** Topic areas covered by questions to trainees and trainers

**Areas covered in questions to trainees & trainers**	**Area covered in questions to trainees only**	**Areas covered in questions to trainers only**
Quality of clinical experience	Satisfaction with the post	Support received in role as clinical supervisor
Accuracy and use of the job description	Absence of bullying and undermining behaviour	
Opportunities in the post for involvement in:	Having to work beyond clinical competence	
a) Research		
b) Teaching		
c) Management		
Quality of clinical environment	Regular observation of work by seniors	
Quality and frequency of Clinical Supervision	Post helpful for achieving Annual Review of Competence Progression (ARCP) competence	

### Interviewers

Two interviewers conducted the interviews with all psychiatry trainees and trainers to minimize inter-rater variability. Both interviewers were consultant psychiatrists with extensive experience in psychiatric education. Training posts in SLaM are spread over more than 120 settings, including six hospitals. Neither of the interviewers conducted interviews at the hospitals in which they worked, nor did they interview any of their past or present trainees. Initially, both interviewers interviewed 32 consultant trainers and 27 trainees at the same time to compare inter-rater variability. The rest of the trainers and trainees were interviewed by one interviewer only thereafter.

### Interview scoring

The interviewers asked trainers and trainees questions within each area in Table 2. Ratings were on a scale of 0 – 3, using a pre-defined criteria. The extensive questionnaires and the corresponding scoring schemes for trainees and trainers are too lengthy to be included within the limited remit of a research article. An example of a scoring scheme used for one of the interview domains (Clinical Supervision) is shown instead in Table 2.

**Table 2:** Scoring scheme used for the interview domain ‘clinical supervision’

**Score**	**Description**
0	No regular supervision by any senior clinician
1	Supervision with trainer on ad hoc basis/no fixed regular slot/content predominantly clinical management
2	Regular supervision but no dedicated time/trainer’s other commitments take priority
3	Dedicated hour every week/protected from other commitments/in trainee’s job plan/plan discussed at start of post

Once the answer was scored by the interviewer in line with the scoring scheme, trainees and trainers were also given the opportunity to give comments about reasons for giving their answers. These comments were recorded separately as free text and did not affect the scoring of the answers.

### Combined interview domains scores

A combined score was generated by calculating the average trainee and trainer score for the interview domains common to both the trainees and trainers. These interview domains were: Job description, clinical opportunities, clinical environment and clinical supervision, research opportunities, teaching opportunities and management opportunities. The reason these scores were combined was to generate an overall score which more accurately reflects the multifaceted nature of each training post. The combined scores were then used to compare the following types of placements:

1. *General Adult and Specialist psychiatry*

b. Specialist psychiatry:

This included all non-general adult psychiatry posts. Examples include: addictions, alcohol and substance misuse, eating disorders, personality disorders, behaviour disorders, affective disorders, obsessive compulsive disorders, learning disabilities, forensic psychiatry, treatment resistant psychosis, child psychiatry, adolescent psychiatry, perinatal psychiatry, autistic disorders, neurodevelopmental psychiatry, neuropsychiatry, psychotherapy, rehabilitation and liaison psychiatry.

b. General adult psychiatry:

This included all non-general adult psychiatry posts.

2. *Specialist and Liaison psychiatry*

a. Liaison psychiatry was separated from specialist psychiatry in this section, and the combined scores of each compared. The range of specialties within liaison psychiatry included: Perinatal psychiatry, neuropsychiatry, old age psychiatry and general hospital liaison psychiatry.

### Statistical tests

Pearson’s correlation was used to test for correlation between interviewers scoring of responses for the same candidates, and to test for correlation between trainee and trainer responses. Cronbach’s Alpha was used to test for consistency between the two interviewers’ scoring for the same interviewees. The student *t*-test was used to analyse for differences between mean interview scores for different groups. Linear regression and ANOVA were used to compare differences in job satisfaction scores between trainees at the three different stages of training.

### Ethical approval

Ethical approval was not obtained for this study, as we did not deem it to be necessary. We carefully studied the local guidance issued by King's College London research ethics committee about what constitutes research versus a teaching evaluation. Further, we used the Medical Research Council (MRC) Health Research Authority (HRA) decision algorithm [14] which deemed that our project did not require ethics approval. Finally we also consulted the NHS National Research Ethics Service guidance on 'Is your project research?’ [15] and we carefully read the detailed guidance on this – defining research which can be found at the above link. This was an audit of our training posts, designed as part of service improvement intended to drive up quality, and one that had no research hypothesis and no intervention. As part of our quality improvement programme, it does not require ethical approval, any more than the annual national trainee survey does. Both trainees and trainers were introduced to the survey via a letter which outlined the provenance and purpose of the work. Participation in the survey was optional. Individual trainees who ask how their data would be used were reassured that their individual scores of posts would only be used internally. Therefore, only aggregates scores were published in this study. One gain from the survey is that it has now provided a rationale and hypothesis for what would be a research project (For example, a future RCT of anonymised versus non-anonymised responses), but that is a matter for the future.

## Results

### Inter-rater correlation and consistency

Initially, 36 consultant trainers and 27 trainees were jointly interviewed by the two consultant interviewers. The average total scores obtained by each interviewer were then compared. The Pearson’s correlation coefficient for the two interviewers was r = 0.74 for trainer scores and r = 0.92 for trainees’ scores. Cronbach’s alpha internal consistency test was α = 0.82 for trainers and α = 0.96 for trainees. A significant correlation and consistency between the two interviewers scores was therefore obtained for the same trainees and trainers.

The Pearson’s correlation coefficient for the combined interview scores obtained by the two interviewers is 0.78 (p< 0.001), and Cronbach’s Alpha is α = 0.87. These results indicate that there is also a significant correlation and consistency between the combined scores obtained by the two interviewers. There was no significant difference between the combined scores of the two interviewers (Two tailed *t*-test, p= 0.422).

### Similarity of trainee and trainer feedback

**Figure 2:**
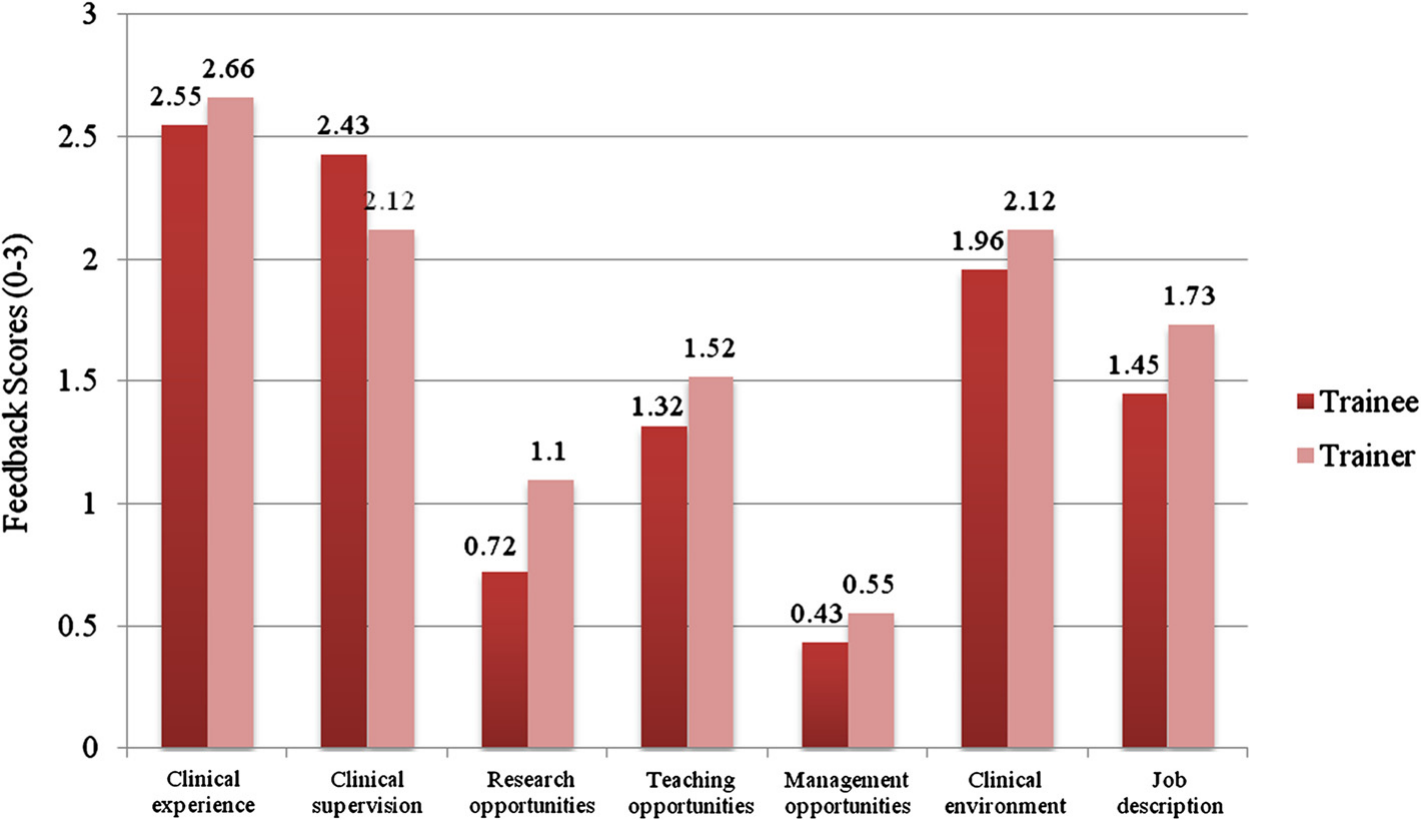
Summary of trainee and trainer feedback results for the same interview areas.

Figure 2 illustrates a summary of the trainee and trainer feedback results on the same domains of training posts.

There is a highly significant correlation between the total average scores of trainees and trainers for the interview domains displayed in Figure 2 (Pearson’s R = 0.968, p< 0.001). Although in general trainers scored each domain higher than trainees, the difference between the average scores for domains by trainees and trainers was not significant (paired samples *t*-test, p = 0.154). This suggests that results obtained from non-anonymised interviews are similar for trainees and trainers.

### General adult and specialist psychiatry

**Figure 3:**
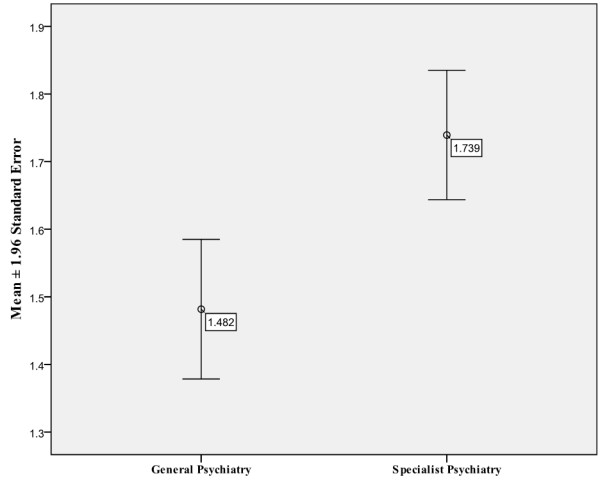
Combined scores of General and Specialist Psychiatry.

**Figure 4:**
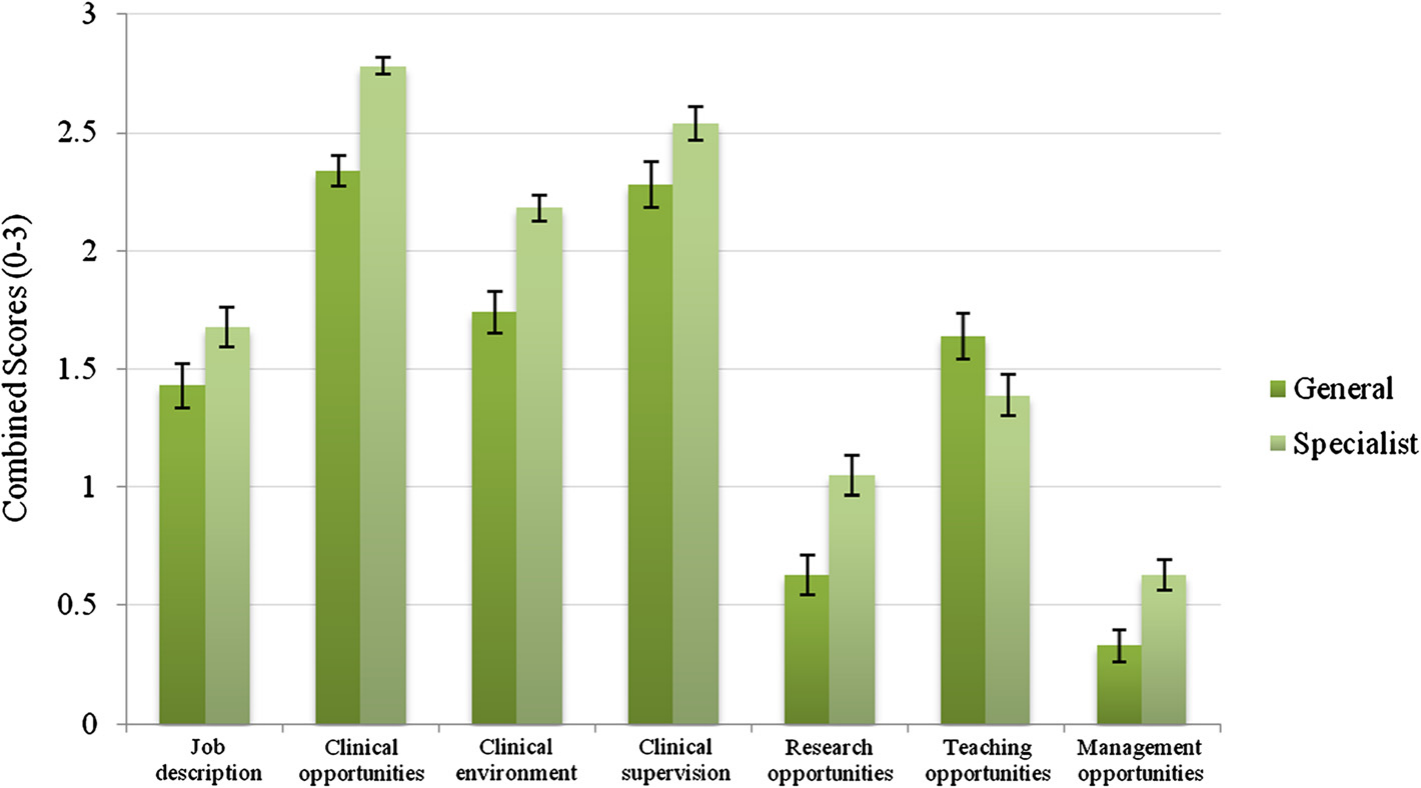
Combined scores of general and specialist psychiatry interview domains.

As Figure 3 shows, the combined score is significantly higher for specialist psychiatry posts (Two tailed *t*-test, p < 0.001, 95% CI: -0.398 to −0.132) than for general adult psychiatry posts. As Figure 4 below illustrates, the interview domains in which the biggest difference was observed between general adult and specialist psychiatry are clinical opportunities and clinical environment (*t*-test, p< 0.001), followed by research opportunities (p= 0.001).

Trainees commented that the busy and stretched clinical environment in general adult psychiatry posts, especially the inpatient posts, was not conducive to trainees getting a good learning experience. Trainees were asked about protective factors which improved their general adult inpatient psychiatry training experience. The factors elicited included a consistently and actively involved consultant or higher trainee, and a balance of a more senior and a junior core trainee working together. A well-established and functional multidisciplinary team also helped to improve the morale and sense of camaraderie within the working place.

### Specialist and liaison psychiatry

**Figure 5:**
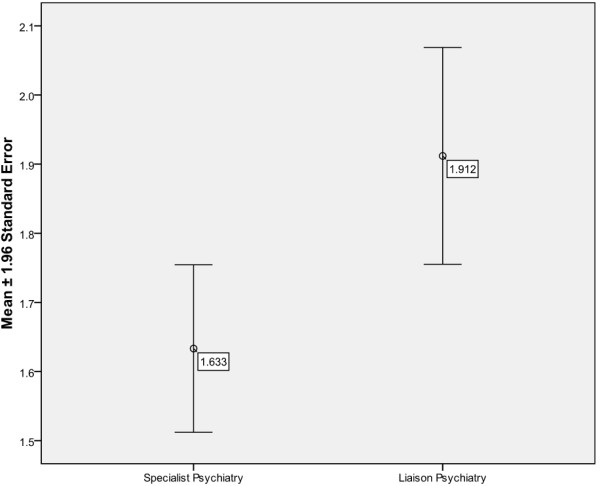
Combined scores of Liaison and Specialist Psychiatry.

As shown in Figure 5, the combined score is significantly higher for liaison psychiatry (*t*-test: p = 0.038, 95% CI: -0.398 to −0.132) than that for specialist psychiatry. Many trainees enjoyed liaison psychiatry as it gave them the opportunity to work in a general hospital setting and interact with clinicians from various clinical specialties. They felt less alienated from the rest of the medical profession and were better supervised.

### Job satisfaction by year of training

**Figure 6:**
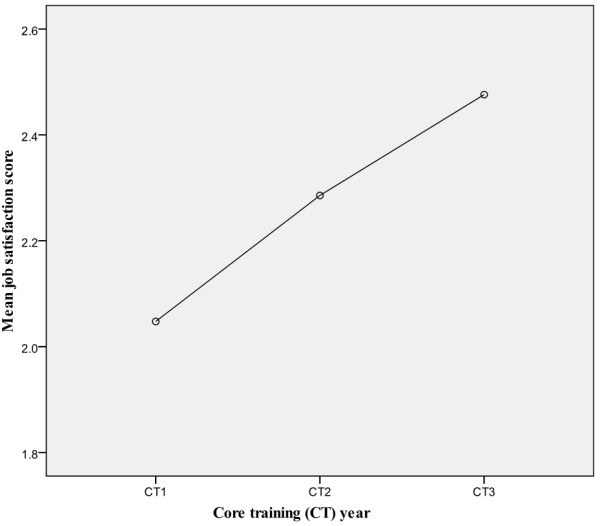
Job Satisfaction by Year of Training.

The majority of trainees were satisfied with their jobs. There was an increasing trend in job satisfaction from year 1 to year 3 of core training (i.e. CT1 to CT3 years) as illustrated in Figure 6.

The linear regression coefficient for job satisfaction scores of core trainees in first, second and third year of training is 0.273 (95% CI: 0.033 to 0.513), and there is a statistically significant difference in job satisfaction scores with increasing seniority (ANOVA p= 0.026). Trainees were then categorised into trainees in General Adult Psychiatry posts or Specialist Psychiatry posts, as shown in Table 3.

**Table 3:** Distribution of trainees in general adult and specialist psychiatry

	**General adult psychiatry**	**Specialist psychiatry**
CT1 trainees	21 (48%)	3 (6%)
CT2 trainees	11 (25%)	21 (41%)
CT3 trainees	12 (27%)	27 (53%)
Total	44	51

There is a greater proportion of junior psychiatry trainees in General Adult Psychiatry, as compared to Specialist Psychiatry, in which there is a greater proportion of senior psychiatry trainees. This may explain the observed difference in job satisfaction scores between CT1, CT2 and CT3 trainees.

Comments from trainees at different levels of training did suggest that the poor morale of first year trainees improved with time and experience. Moreover, a number of trainees remarked that difficult or even terrible posts in which they were placed in as junior trainees were in retrospect a positive learning experience. This was reported more frequently amongst trainees who have passed their college membership exams.

### Experience and opportunities on the clinical placements

Overall, both trainees and their consultant trainers identified a wide range of high quality clinical opportunities and rated the overall clinical experience on the rotation highly, as shown in Figure 2. Consultant trainers expressed concern that the clinical experience was compromised by the clinical environment and the impositions of the European Working Time Directive. While the majority of trainees received excellent supervision, the main concerns reported by trainees were lack of a regular fixed slot for supervision and the challenges of competing clinical demands. Both trainees and trainers reported very few research opportunities as part of their training posts.

The Maudsley rotation is usually seen as particularly research friendly. Yet despite that, the majority of trainers felt that it was unrealistic to expect their core trainees to undertake research in light of clinical and educational commitments, and many trainees identified the demands of clinical workload, administrative responsibilities and exam preparation as obstacles to involvement in research. However, on the more optimistic side, trainers of academic clinical fellows (ACFs) were notably more supportive of trainees strongly participating in research, suggesting that the NIHR Integrated Academic Training programme is both necessary and valued.

The main deterrents to involvement in teaching were the arbitrary allocation of medical students with clinical firms and the lack of structured teaching programmes which trainees can get involved in.

Most trainees and trainers reported that as a trainee, there is very limited exposure or active involvement in medical management. A great proportion of trainers cited that such experience is more appropriate for senior trainees. Both trainees and trainers stated that multidisciplinary team meetings are important in maintaining trainees’ awareness of important managerial issues.

## Discussion

Both trainees and their trainers had similar views about the strengths and weaknesses of each post. This study has shown that trainees and trainers report an overall significantly better training experience in specialist psychiatry posts as compared to general adult psychiatry posts. Liaison psychiatry was further shown to be especially popular amongst psychiatry trainees. More senior trainees were significantly more satisfied with their jobs than junior trainees.

### Specialist and general adult psychiatry

A significant number of trainees in general adult inpatient psychiatry training posts were experiencing difficulty due to aggression from patients, rapid patient turnover, limited contact with patients and the extensive paperwork (e.g. discharge summaries) and the lack of acceptable facilities, including computer access or functioning ECG machines. An important frequent remark which came out in interviews was that the role of the trainees in general adult psychiatry placements was entirely dictated by service demands, often with little or no focus on training requirements. The training experience was further compromised by inadequate clinical environments (e.g. lacking enough office space). Many wards seemed to be operating with what might be described as a ‘siege mentality’, with high levels of violence and disturbance, combined with a culture of demoralisation and the sense that both medical staff and nursing staff were feeling impotent, overwhelmed and unable to change the situation. These factors have been previously shown to be associated with high dissatisfaction rates from psychiatry trainees and contributing to decisions to leaving psychiatry [3,4,7]. The authors are not aware of any evidence of educational value in the ‘baptism of fire’ experienced by trainees exposed to such environments and the concept of learning by being ‘thrown in at the deep end’. Some trainees did defend this idea, albeit retrospectively. However, it was more common for trainers to argue that exposure to challenging and unpleasant inpatient settings is beneficial to trainees in their early years of training. Interestingly, most CT1 trainees occupied General Adult psychiatry posts, and very few were allocated to Specialist psychiatry.

### Specificity of feedback

Previous surveys, such as the annual National Trainee Survey, the annual local survey organised by trainees and the annual survey run by the local Deanery are all useful for pointing out general areas of strengths and relative weaknesses. While all of these surveys usefully identified areas of concern, it was not possible to identify and address these areas of concerns at the level of individual placements. On the other hand, the results from this interview study has generated results that have allowed for substantial improvements in training provision and management at the level of each individual training placement on this rotation, which is the main unit of intervention. A strong correlation was shown between the feedback that trainers and trainees gave about remarkably different aspects of each job (e.g. clinical opportunities, research, teaching and management opportunities). This reinforces the ability of a non-anonymised interview scheme with trainees and their trainers in accurately elucidating the positive and negative aspects of their respective training posts. Using a combined score of feedback from both trainer and trainee is likely to be more accurate than relying on an individual trainee or trainer (with the bias that those individual scores may harbour). Because of this, it is possible that this data may be taken more seriously by those responsible for training provision and thus result in changes to improve the training experience of individual posts. However, it could also be argued that the trainers’ awareness of the negative training aspects of the post experienced by their trainees suggests that there may be insurmountable barriers to improving the post structure and experience.

### Job satisfaction by year of training

The reported linear regression and ANOVA test results suggest that there is a significant increase in job satisfaction with increasing seniority. Because the present study is a cross-sectional interview scheme, it cannot be confirmed whether or not job satisfaction truly does increase with increasing seniority. Increasing satisfaction with increasing seniority has however also been suggested in other published trainee surveys [7,16]. Interestingly, the 2011 National Trainee Survey of UK core psychiatry trainees similarly demonstrates an upward trend in trainee satisfaction rates from CT1 to CT3 year, with a higher satisfaction rate in CT3 trainees as compared to CT1 trainees [16]. In our training scheme, as is the case in most psychiatry training programmes, most trainees occupying General Adult Psychiatry posts are CT1 trainees, while most trainees in Specialist Psychiatry posts are CT3 trainees. This may explain the existence of an increasing trend in job satisfaction with increasing seniority. The small group numbers (e.g. 3 out of 51 specialist psychiatry posts are occupied by CT1 trainees), means it is difficult to categorise CT1-CT3 trainees into those in General Adult psychiatry or Specialist psychiatry posts trainees and analyse for a statistically significant difference in job satisfaction levels. Other possible reasons for the increasing trend in job satisfaction are a steep learning curve resulting in higher satisfaction rates in more confident trainees towards the higher end of the curve, or successful completion of MRCPsych examinations.

### Interviews versus questionnaires: implications for future research

Conducting interviews rather than sending out a questionnaire was chosen as a method of training evaluation because it was felt that this method would make trainees and trainers more likely to participate. Indeed this method may be a key factor behind the high response rate from trainees and trainers. Interviewees may feel much more likely to be listened to if they are interviewed rather than having to fill out a questionnaire. An interviewer is able to assess the training environment as well as interviewing the subject about their training experience. Furthermore, interviewers can choose to further explore specific issues highlighted by the interviewee, which would have otherwise remained vague in a questionnaire response. If a confiding relationship is established with the interviewee, more information may be acquired regarding the training post, albeit the opposite may hold true. As far as we know, there is no evidence in the literature indicating if students or trainees are more likely to participate in questionnaires or interviews about their training. This study suggests that assessing training via a non-compulsory interviews scheme may have a high participation rate amongst both trainees and trainers. It would be useful to compare response rates for interviews and questionnaires in future research, especially as the former is potentially more expensive, labour-intensive and time-consuming. Another key question which remains unanswered is which of the two methods of training assessment is more likely to bring about positive and long-term changes to training. It is too early to say if the changes that are being implemented as a result of this survey will make a long term difference. Further research may indeed find one method to be superior to the next. In this study, face to face interviews gave an opportunity to collect rich qualitative data about posts which questionnaires do not collect. Interviews also allowed for discussion and reflection during data collection. Ultimately, the most robust way of settling the risks and benefits of interviews versus questionnaires (other than the self-evident issue of cost) is via a randomized controlled trial (RCT), which might be a future research possibility.

### Strengths of the study

A major strength of this study is the large number of subjects interviewed; 99 trainees and 109 trainers. The response rate by both trainees and trainers for the current study was high at 88% and 97% respectively. The trainee response rate is comparable to the 2011 National Trainee Survey response rate, which was 87% [17], even though the latter is compulsory. On the other hand, our consultant trainers response rate is substantially higher than the 2011 National Trainer Survey response rate, which was 43.3% [18]. However, we do not know if such high responses would be maintained over time, as “survey fatigue” might set in. The high response rates suggest that non-anonymising of data is not a deterrent to trainees and trainers giving detailed feedback about their training posts. The advantage of non-anonymising of data is that it allows multi-factorial aspects of each particular training post to come to the surface. It may act as an incentive to participants because they may feel that their responses are more likely to bring specific training issues to light, and bring about change to their respective posts. The high response may however be a limitation of this study, as will be discussed in the next section.

### Limitations of the study

There are a number of limitations to this study. Firstly, although it has been stated that the high response rate is a strength because it indicates willingness by trainees and trainers to participate in a non-anonymised interview scheme, it may conversely be a limitation of this study. Although participation was optional and not compulsory, trainees and trainers may have felt pressured by the fact that they were emailed by the vice dean for education and training requesting them to participate in the interview scheme. This may explain the high trainee response rate. Struggling trainees may feel obliged to participate to avoid further training problems, and may not give true feedback if they feel that their responses will be linked to them. As a matter of fact, this may apply to all trainees, not only struggling ones. This certainly was not the impression of the interviewers, and nor has such a suggestion surfaced from any trainee in the two years that have passed since the study was completed. Instead, it has been our impression that the survey has had a positive effect on morale, indicating a desire to get detailed feedback and information at the level of individual posts, rather than the aggregated data from national surveys, which in anything other than a small rotation, is almost impossible to translate into practical action. Another potential weakness of non-anonymised feedback is that trainees in difficulty may be more likely to avoid giving feedback all together, giving a skewed picture of the overall quality of training. In order for this exercise to provide an accurate and non-biased reflection of the quality of training, it is vital that all trainees feel that they can both participate and speak honestly about their training posts. This is why non-anonymised feedback may best serve as an additional training assessment tool, rather than an alternative to anonymised feedback. The advantages and disadvantages of non-anonymised feedback in training evaluation is certainly an area which would benefit from further research.

A further limitation of this interview scheme is that it gave a ‘snapshot’ of the training scheme. Feedback about individual training posts may be influenced by individual trainees’ perception, circumstances or relationship with trainers. Results relating to particular training posts are less prone to bias in a rolling trainer and trainee feedback scheme. Another limitation is that interviewers scoring the responses may have personal biases. None of the interviewers interviewed at hospitals in which they worked, however, and nor did they interview their own trainees or consultants they worked with. Interviewing both trainees and trainers, interviewing together for a proportion of the interviews, as well as the assessment of the clinical environment by the interviewers, may have helped limit personal biases. The interviews were carried out by two consultant psychiatrists, making this interview scheme a more expensive and labour-intensive method of gathering feedback than online surveys. However, as the training programme assessed in this study is very large, the presented interview scheme would be significantly easier to implement in the average sized training programme.

## Conclusion

This in-depth examination of the quality of training on a large psychiatry training programme successfully elicited strengths and weakness of our programme. Additionally, trends in quality of training and job satisfaction amongst various psychiatric specialities were identified. Such an interview scheme could be easily implemented in smaller schemes and may well provide important information to allow for targeted improvement of training.

## Competing interests

All of the authors declare that they have no competing interests.

## Authors’ contributions

MB analysed and interpreted the data, performed the statistical analysis and wrote the manuscript. EI and EP participated in the design of the study, performed all of the interviews and helped interpret the data and draft the manuscript. JR helped interpret the data and draft the manuscript. AI and SW conceived of the study, participated in its design and helped interpret the data and draft the manuscript. All authors read and approved the final manuscript.

## Authors’ information

Mukhtar Bizrah is an Academic Foundation Programme Doctor at Guy’s and St Thomas’ Hospitals NHS Foundation Trust and King’s College London (KCL) Division of Medical Education. His research focus is in Postgraduate Medical Education, and he has a strong interest in teaching.

Eduardo Iacoponi obtained his PhD in Epidemiology from the Institute of Psychiatry, KCL. As Clinical Adviser to KCL School of Medicine and Educational Supervisor to the Maudsley Psychiatry rotation, he has been actively involved in the training of medical students and psychiatrists for the past 20 years.

Elizabeth Parker is a Consultant Psychiatrist with the South London and Maudsley NHS Foundation Trust (SLaM) and retired Training Programme Director for the Guys and St Thomas' Higher Training Scheme in General and Old Age Psychiatry.

Janice Rymer is Dean of Undergraduate Medical Education, Chairman of Medical Education and Professor of Gynaecology at KCL. She is an educationalist supervisor for specialist trainees and a preceptor for specialist advanced training modules in medical education. She has published over 130 peer-reviewed research papers and 15 textbooks.

Amy Iversen is a Consultant Liaison Psychiatrist and a Senior Lecturer in Medical Education at the Institute of Psychiatry, KCL. She has a special interest in interventions to improve the quality of psychiatry training at undergraduate and post-graduate level.

Simon Wessely is Chair and Head of the Department of Psychological Medicine at the Institute of Psychiatry, KCL. He is a consultation liaison psychiatrist, and is also trained in epidemiology. As Vice Dean for Academic Psychiatry he is committed to promoting psychiatry and improving clinical and academic training.

Dr Amy Iversen and Professor Sir Simon Wessely are joint last authors.

## Pre-publication history

The pre-publication history for this paper can be accessed here:

http://www.biomedcentral.com/1472-6920/13/85/prepub

## References

[B1] Cooper B British psychiatry and its discontents JRSM 2010 103 10 397 402 10.1258/jrsm.2010.100041 PMC2951176 20929890

[B2] Brown N Vassilas CA Oakley C Recruiting psychiatrists – a Sisyphean task? Psychiatr Bull 2009 33 10 390 392 10.1192/pb.bp.108.024638

[B3] Lambert TW Turner G Fazel S Goldacre MJ Reasons why some UK medical graduates who initially choose psychiatry do not pursue it as a long-term career Psychol Med 2006 36 5 679 684 10.1017/S0033291705007038 16426488

[B4] Rowan WC Atkins CL Marston G M Trainee views on active problems and issues in UK psychiatry: Regions Collegiate Trainees' Committee survey of three UK training Psychiatr Bull 2000 24 336 338 10.1192/pb.24.9.336

[B5] Gomez-Beneyto M Montilla-Garcia JF de Castro-Manglano P Gay-Pamos E Gonzalez-Torres MA Gutierrez-Fraile M Kuhalainen-Munar A Lopez-Ibor JJ Marquez-Gallego F Peralta-Martin V The opinion of psychiatric residents on the training they receive Actas Esp Psiquiatr 2011 39 3 174 179 21560078

[B6] Giaglis G Angelidis G A questionnaire on Satisfaction from psychiatric Training Psychiatrike 2008 19 1 35 42 22217814

[B7] Barras C Harris J Psychiatry recruited you, but will it retain you? Survey of trainees’ opinions Psychiatrist 2012 36 2 71 77 10.1192/pb.bp.111.034413

[B8] GMC National Training Surveys reports [http://gmc-onlineeducationreports.org]

[B9] Rickenbach M Wedderburn C The PMETB National Trainee Survey: is it a useful tool? Educ Prim Care 2010 21 2 72 75 20359383 10.1080/14739879.2010.11493883

[B10] Smith D Hamilton-Fairley D Has the national survey of trainee doctors improved training in the UK? Br J Hosp Med (Lond) 2011 72 8 424 425 21841583 10.12968/hmed.2011.72.8.424

[B11] The Maudsley Training Programme London, UK [http://www.maudsleytraining.com]

[B12] South London and Maudsley NHS Foundation Trust (SLaM) London, UK [http://www.slam.nhs.uk]

[B13] GMC National Training Survey Report 2010 [http://www.gmc-uk.org/static/documents/content/Training_survey-FINAL2010.pdf]

[B14] Is my study research? [http://www.hra-decisiontools.org.uk/research]

[B15] Is your project research? [http://www.nres.nhs.uk/applications/is-your-project-research/]

[B16] National Trainee Survey 2011 [http://gmc-onlineeducationreports.org/IndicatorScores.aspx?agg=AGG27%7c2011&groupcluster=S91]

[B17] GMC National Survey of Trainees 2011 [http://www.gmc-uk.org/NTS_trainee_survey_2011.pdf_45270429.pdf]

[B18] GMC National Survey of Trainers 2011 [http://www.gmc-uk.org/National_Trainer_survey_2011_v5.pdf_48076220.pdf]

